# Iatrogenic adrenaline induced mid-ventricular Takotsubo cardiomyopathy: a case-based review

**DOI:** 10.1007/s11845-022-03000-2

**Published:** 2022-04-09

**Authors:** Adam Ioannou

**Affiliations:** Department of Cardiology, Royal Free Hospital, Royal Free NHS Foundation Trust, Pond Street, London, NW3 2QG UK

**Keywords:** Adrenaline, Cardiac magnetic resonance imaging, Catecholamine toxicity, Mid-ventricular Takotsubo cardiomyopathy, Takotsubo cardiomyopathy

## Abstract

Takotsubo cardiomyopathy (TCM) is regarded as an acute and often reversible cardiac syndrome characterised by apical ballooning of the left ventricle that occurs in the absence of coronary artery obstruction and myocarditis. The underlying pathophysiology remains largely unknown, but the most widely accepted theory is catecholamine toxicity.

More recently, atypical variants of TCM have been described, and are characterised by the regional wall motion abnormalities that are observed. Mid-ventricular Takotsubo cardiomyopathy (MVTCM) is characterised by hypokinesia/akinesia of the mid left ventricular wall segments with hyperdynamic basal and apical function. This report describes the first documented case of a patient who developed MVTCM after receiving a dose of intravenous adrenaline. This case provides further evidence to support the notion that catecholamine toxicity is implicated in the pathogenesis of TCM.

## Introduction

**Fig. 1:**
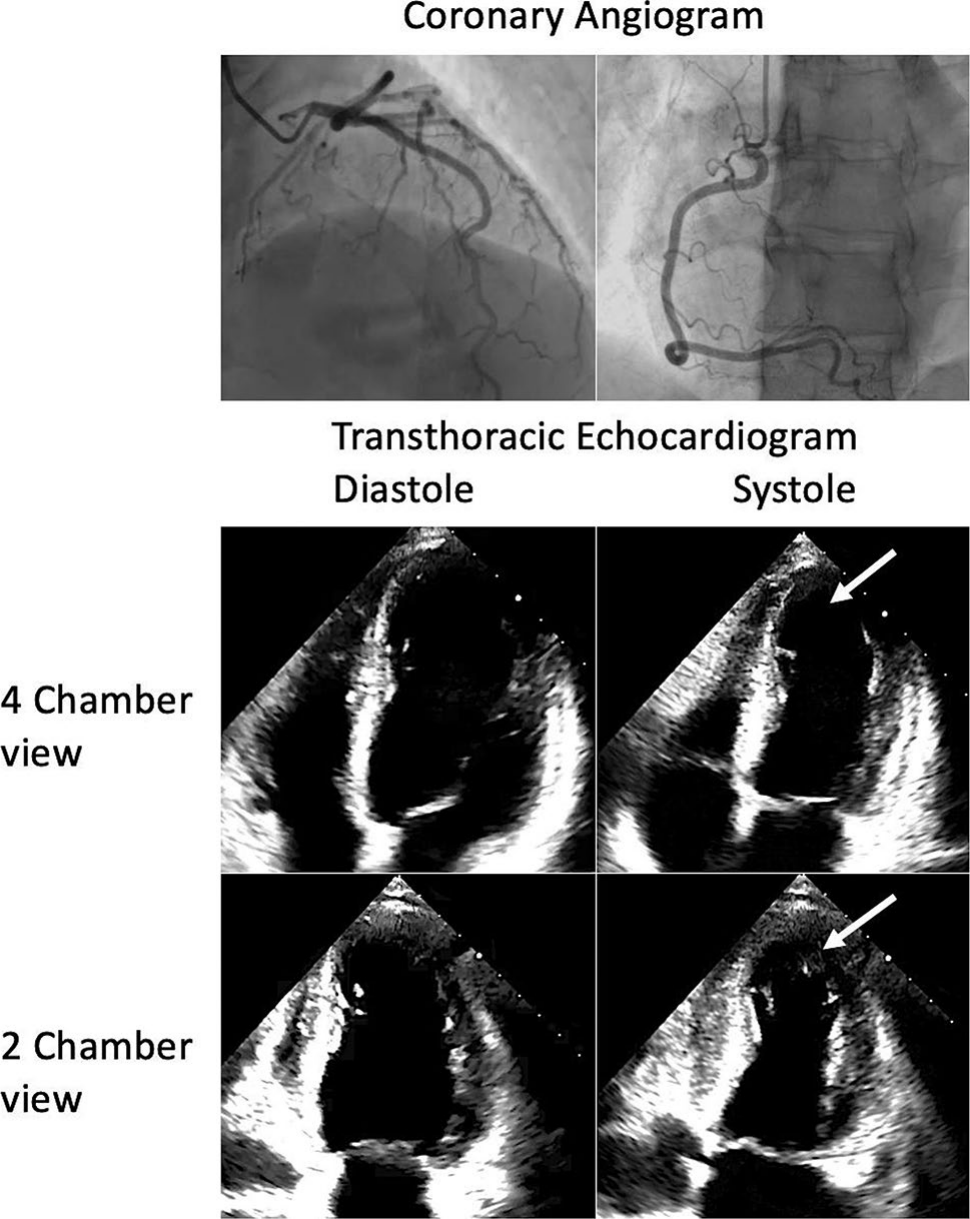
Unobstructed coronary arteries seen on a coronary angiogram (top panel) and a transthoracic echocardiogram demonstrating apical akinesis (white arrows). These findings confirm a diagnosis of conventional apical Takotsubo cardiomyopathy

Classically, Takotsubo cardiomyopathy (TCM) is regarded as an acute and often reversible cardiac syndrome characterised by apical ballooning of the left ventricle. It often occurs as a result of intense emotional or physical stress, and hence is commonly referred to as stress cardiomyopathy or ‘broken heart syndrome’. The underlying pathophysiology remains largely unknown, but various theories have been proposed such as coronary artery spasm and coronary microvascular disease; but the most widely accepted theory is catecholamine toxicity [1]. A diagnosis of TCM must fulfil the following criteria: regional wall motion abnormalities, unobstructed coronaries, elevated troponin and the absence of active myocarditis [2]. Since the discovery of TCM, various different subtypes have been described. Presentations can be broadly categorised into two main groups: Typical TCM which involves apical regional wall motion abnormalities (Fig. 1), and atypical TCM which involves non-apical regional wall motion abnormalities such as left ventricular basal or mid wall hypokinesis/akinesis [3] or even isolated right ventricular ballooning [4]. The categorisation of each atypical subtype depends on which regional wall motion abnormalities are observed. Mid-ventricular Takotsubo cardiomyopathy (MVTCM) is characterised by hypokinesia/akinesia of the mid left ventricular wall segments with hyperdynamic basal and apical function [5]. This report describes a patient who developed MVTCM after receiving a dose of intravenous adrenaline.

## Case report

A young lady who was 39 weeks pregnant developed post-partum haemorrhage following a vaginal delivery. She was immediately transfused with 4 units of cross-matched blood. During this period, a venous blood gas demonstrated a low serum calcium. The anaesthetist attempted to replace this with intravenous calcium gluconate but accidentally drew up and administered a bolus of intravenous adrenaline. Immediately following this, the patient had a cardiac arrest. The initial rhythm was ventricular fibrillation, and following a single direct current cardioversion shock converted to pulseless electrical activity. Return of spontaneous circulation was achieved after 4 cycles of cardiopulmonary resuscitation. The patient was then transferred to intensive care where endotracheal ventilation continued and the patient received haemodynamic support with noradrenaline and dobutamine.

**Fig. 2:**
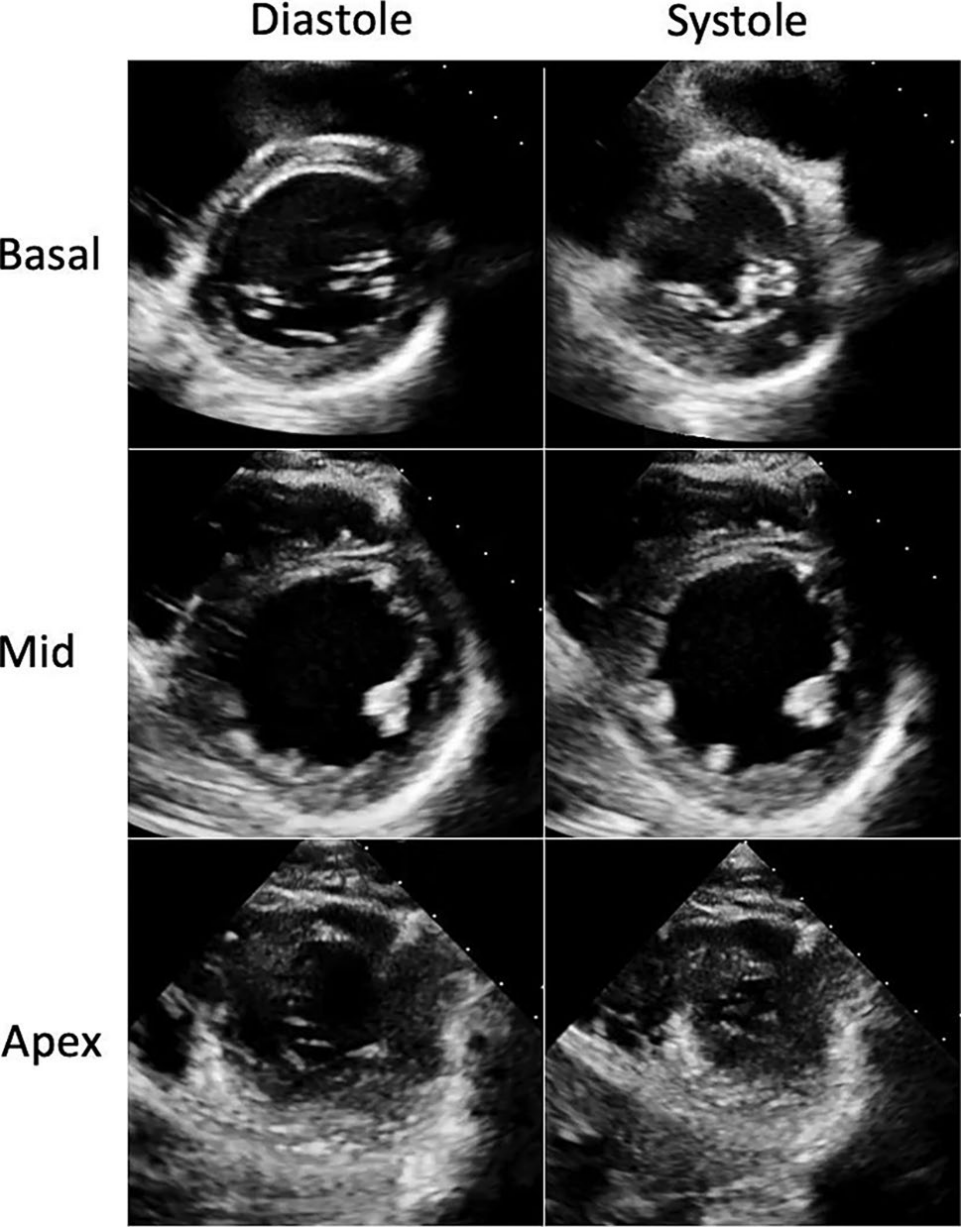
Transthoracic echocardiogram parasternal short axis views of the left ventricle demonstrating circumferential mid ventricular akinesis

Initial blood tests demonstrated a troponin-T 6261 ng/L, which then decreased to 3784 ng/L 24 h later. All remaining routine biochemistry and haematological blood results were unremarkable. A resting 12-lead electrocardiogram showed a sinus tachycardia with no ischaemic changes and a normal QTc. A bedside transthoracic echocardiogram demonstrated a dilated left ventricular cavity with moderately impaired systolic function (ejection fraction = 39% [calculated using the Simpson’s biplane method]). The mid-left ventricular segments were akinetic, while the basal and apical segments were hyperdynamic (Fig. 2).

**Fig. 3:**
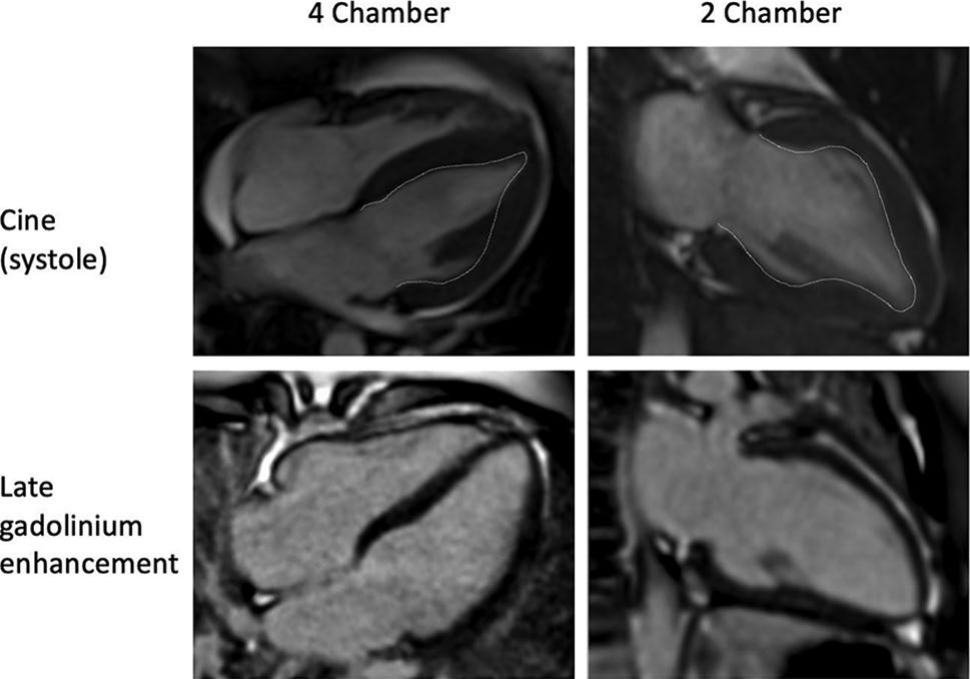
Cardiac magnetic resonance imaging demonstrating mid ventricular hypokinesis (with the endocardial border being highlighted [top panel]), and no late gadolinium enhancement (bottom panel)

The patient made a good recovery, and after 48 h was extubated and weaned off of haemodynamic support. Cardiac magnetic resonance imaging demonstrated that the mid anterior, inferior, lateral and septal segments were mildly hypokinetic, and the left ventricular systolic function improved significantly (ejection fraction = 49%). There were elevated native-T1 and T2 mapping signals seen in the mid left ventricular segments, consistent with myocardial oedema, but there was no late gadolinium enhancement to indicate infarction, scar or fibrosis (Fig. 3). The patient remained haemodynamically stable and was discharged home 1 week after the initial cardiac arrest.

A follow-up cardiac magnetic resonance imaging scan 3 months later demonstrated complete recovery of her left ventricular systolic function. There were no regional wall motion abnormalities, and on tissue characterisation native-T1 and T2 values returned to within normal limits, and there was no late gadolinium enhancement.

The clinical history of accidental adrenaline administration, troponin rise, transient moderate left ventricular systolic impairment and myocardial oedema in the mid segments is consistent with a diagnosis of MVTCM.

## Discussion

TCM accounts for 1–2% of patients who present to hospital with an acute coronary syndrome; and hence the initial diagnosis and subsequent treatment of these patients can be challenging. MVTCM is the second most common form of TCM, and accounts for roughly 15% of cases; compared to the far more common conventional apical TCM, which accounts for 82% of cases. The majority of patients present with chest pain, while the second most common presenting complaint is shortness of breath. MVTCM is far more common in women, the majority of whom are postmenopausal. Initial investigations usually reveal raised cardiac enzymes, and the resting electrocardiogram may demonstrate ST-changes, while the QTc can be markedly prolonged [5, 6].

The underlying triggers for MVTCM are reported to be similar to those for conventional apical TCM, with many patients presenting after significant emotional or physical stress. However, in over one quarter of patients, a trigger is often not identified [6]. The above case describes a patient who developed MVTCM following an accidental bolus of adrenaline. Conventional apical TCM and reverse TCM (a variant that leads to basal hypokinesis/akinesis and a hyperdynamic apex) have been previously described following the use of adrenaline, but this appears to be the first case of iatrogenic MVTCM precipitated by an adrenaline bolus [7, 8]. The development of a reversible stress cardiomyopathy has also been associated with an underlying catecholamine secreting phaeochromocytoma [9, 10]. Additional precipitants include methamphetamine and excessive energy drink consumption, which are thought to cause increased sympathetic drive and may mimic the pathophysiology caused by extreme catecholaminergic activity [11, 12]. These cases provide further weight to the theory that TCM and its multiple variants are precipitated by catecholamine toxicity.

In order to exclude acute coronary syndrome and confirm a diagnosis of TCM, patients previously required an invasive coronary angiogram as part of their work up; however, the more recent emergence of advanced cardiac imaging techniques has resulted in clinicians combining the clinical history with cardiac imaging to establish a diagnosis, rather than proceeding straight to an invasive angiogram. Cardiac magnetic resonance imaging has emerged as a first line diagnostic tool in suspected cases of TCM, and was utilised in the case described. The use of steady state-free precession cine imaging is used to identify regional wall motion abnormalities, while black-blood T2-weighted triple inversion recovery can be used to identify myocardial oedema, and 2D inversion-recovery gradient echo 5 − 10 min after gadolinium contrast administration is utilised to identify areas of myocardial necrosis. Typically, T2-weighted imaging reveals circumferential transmural myocardial oedema matching the regional wall motion abnormalities [13].

Early cardiac magnetic resonance imaging studies suggested that the lack of late gadolinium enhancement was necessary to diagnose TCM, as its presence would suggest irreversible myocardial necrosis caused by an alternative underlying disease process. It was previously thought that the presence of late gadolinium enhancement could help differentiate acute myocardial infarction and myocarditis from TCM [14]. However, more recent studies have shown that late gadolinium enhancement can occur in TCM, and is associated with a poor prognosis [15]. Subsequent experiments demonstrated that delayed washout of gadolinium may be caused by increased interstitial water content associated with transient myocardial oedema, rather than irreversible myocardial necrosis. Hence, the presence of late gadolinium enhancement may reflect the severity of the underlying myopathic process, rather than the presence of scar [16]. However, this does produce a significant diagnostic challenge, as the presence of late gadolinium enhancement raises the suspicion of an alternative pathology such as myocardial infarction or myocarditis. In view of the case described, it would be reasonable to consider cardiac magnetic resonance imaging as a first line investigation in cases where there is a strong clinical suspicion of TCM and no risk factors for coronary artery disease. If the imaging confirms characteristic findings of TCM, without any late gadolinium enhancement, then an invasive coronary angiogram could be avoided. However, if there is late gadolinium enhancement, it is likely that the patient will need an angiogram to exclude an acute coronary syndrome.

Often, a diagnosis can be established following a multiparametric approach that utilises either cardiac magnetic resonance imaging or a combination of transthoracic echocardiography and an invasive coronary angiogram. Rarely, there are cases where there is diagnostic uncertainty following the above investigations, and once ischaemia has been excluded through coronary angiography, an endomyocardial biopsy may be required to differentiate between TCM and myocarditis. Endomyocardial biopsy is likely to demonstrate features consistent with a myopathic process such as myocyte hypertrophy with enlarged nuclei and nucleoli [11]. In order to establish a diagnosis of TCM on endomyocardial biopsy, the presence of myocarditis must be excluded; and hence there must be no histological evidence of myocardial inflammatory infiltrate or myocyte degeneration/necrosis [17].

Management of TCM is predominantly supportive. There is ongoing debate amongst cardiologists regarding the use of conventional heart failure medications such as beta-blocker and angiotensin converter enzyme inhibitors in this cohort of patients, but at present there is no concrete evidence to suggest that their use is beneficial [18]. In a large international registry of TCM patients, the use of angiotensin converter enzyme inhibitors but not beta-blockers was associated with improved survival. Nonetheless, due to the proposed mechanism of catecholamine toxicity provoking TCM, it has been hypothesized that beta-blocker use may prevent recurrence; but in the same registry, over half of the patients with recurrent TCM were taking beta-blockers prior to their second event [6]. In the case described above, our patient was initially in cardiogenic shock and therefore would not have tolerated the initiation of heart failure medications. Once her blood pressure had stabilized, repeat cardiac imaging demonstrated almost complete recovery of her left ventricular function, and therefore it was felt that heart failure medications were unlikely to be of benefit.

Although TCM and its variants are regarded as reversible cardiomyopathies, patients are still at a significant risk of morbidity and mortality. Complications include left ventricular thrombus formation, ischaemic stroke, ventricular tachycardia and cardiogenic shock, such as in the case outlined above [6, 11]. This case serves as a reminder that patients with MVTCM are at significant risk of cardiogenic shock and although it was classically thought to be a relatively benign condition, clinicians must remain vigilant due to a significant risk of complications. Those who develop cardiogenic shock often respond well to a short duration of inotropic support; but in those with a suboptimal response, clinicians should consider transfer to a tertiary cardiac center with the facility to provide extracorporeal membranous oxygenation [11, 18].

In summary, this case describes a patient who developed MVTCM following an intravenous adrenaline bolus. This is the first case of iatrogenic MVTCM, and provides further evidence to support the notion that catecholamine toxicity is implicated in the pathogenesis of TCM. The combination of a clear precipitant and characteristic cardiac magnetic resonance imaging findings meant that a diagnosis could be established without the need for an invasive coronary angiogram; and serial imaging confirmed complete recovery of the left ventricular systolic function. Lastly, although patients with MVTCM often have a good prognosis, clinicians must be mindful that complications can occur, and cardiogenic shock should be treated with prompt initiation of inotropes.
